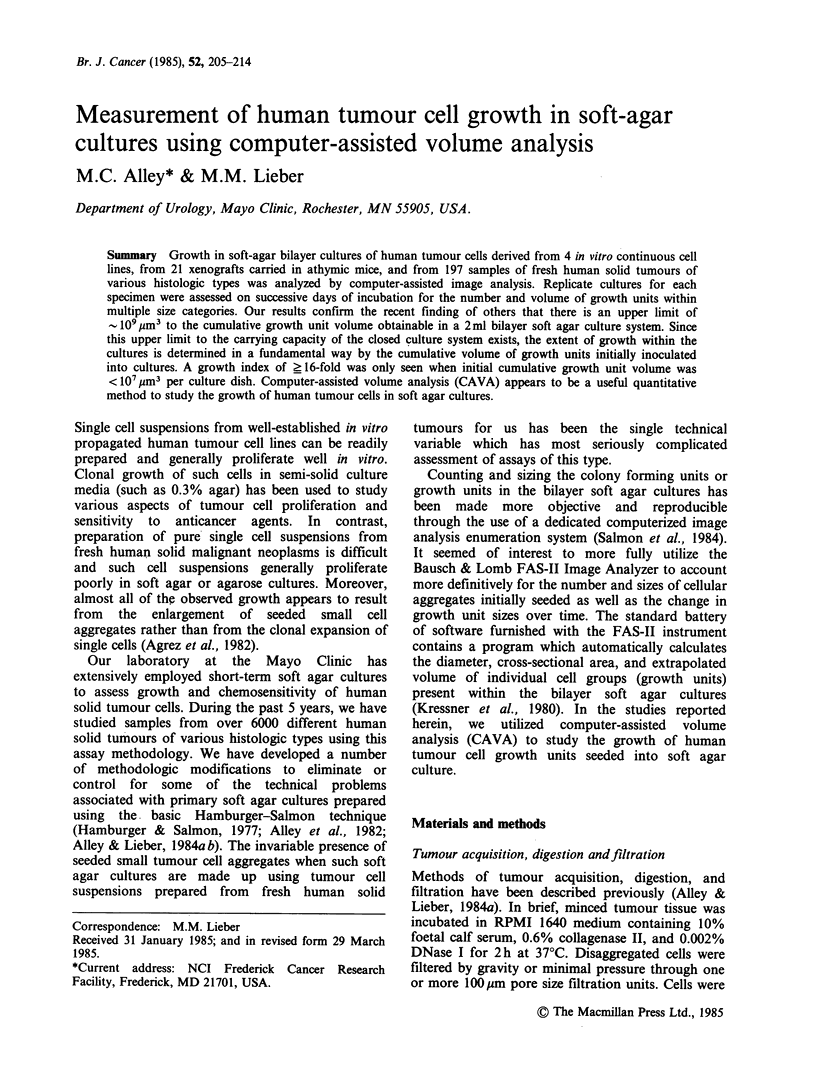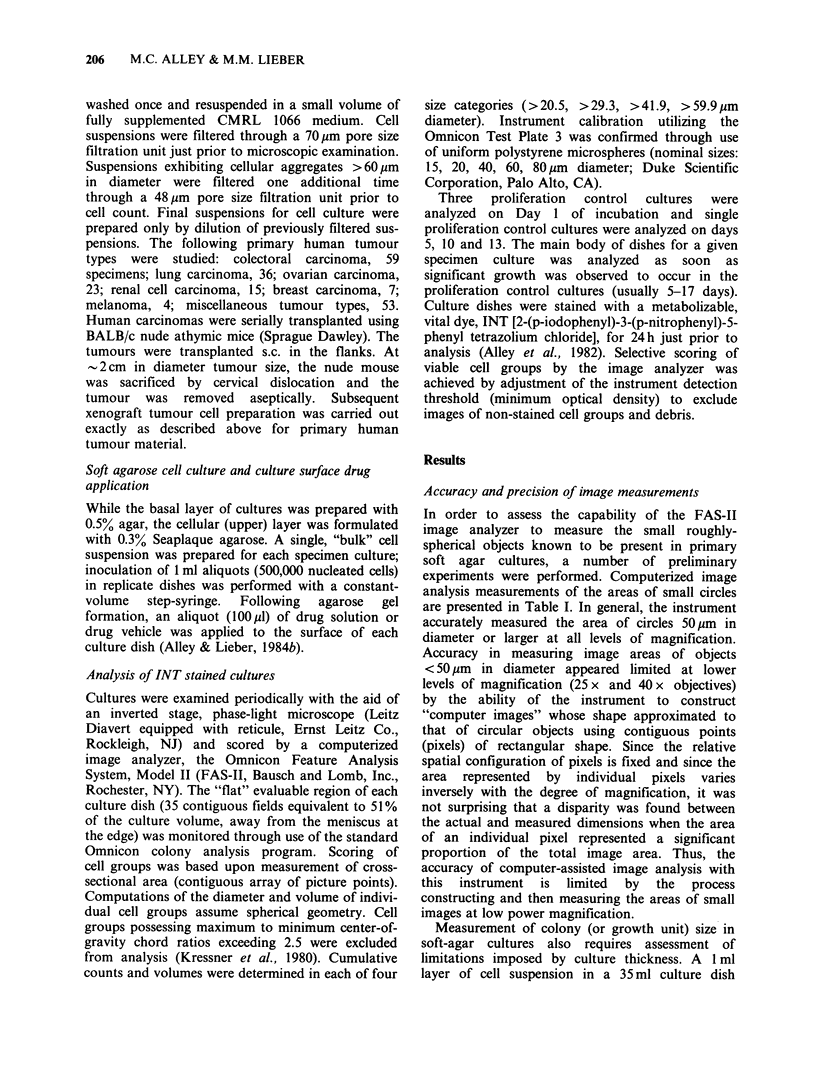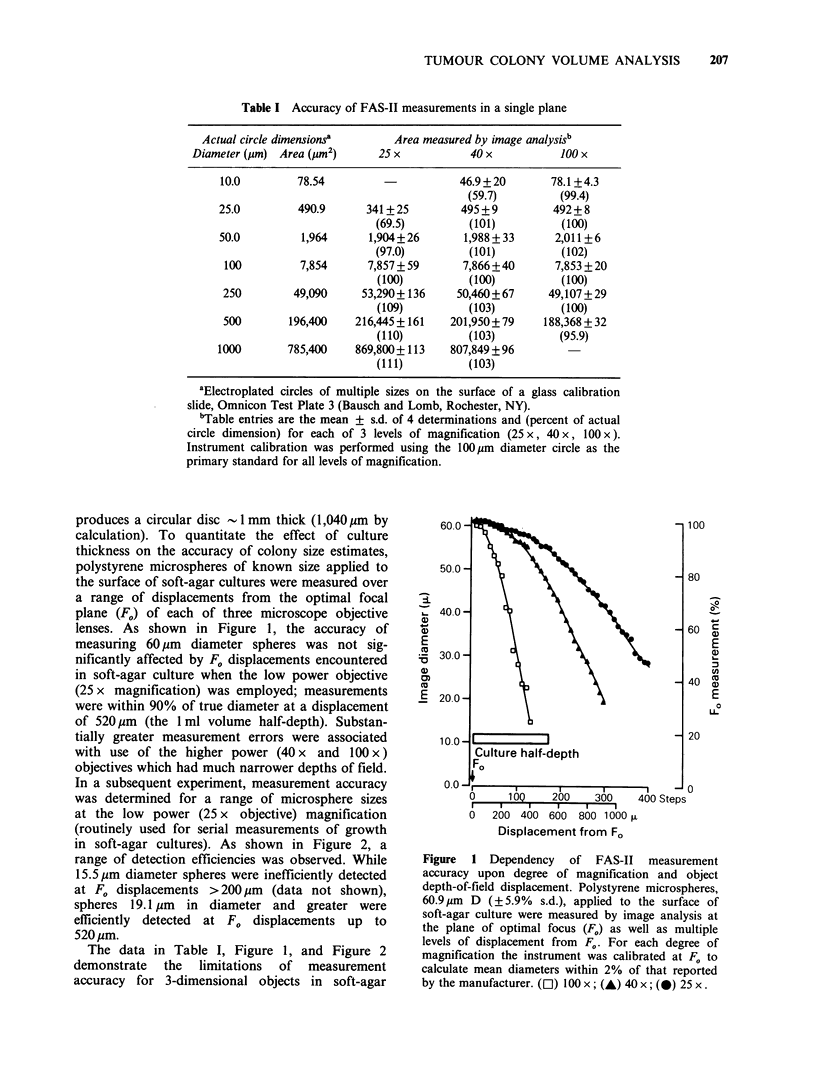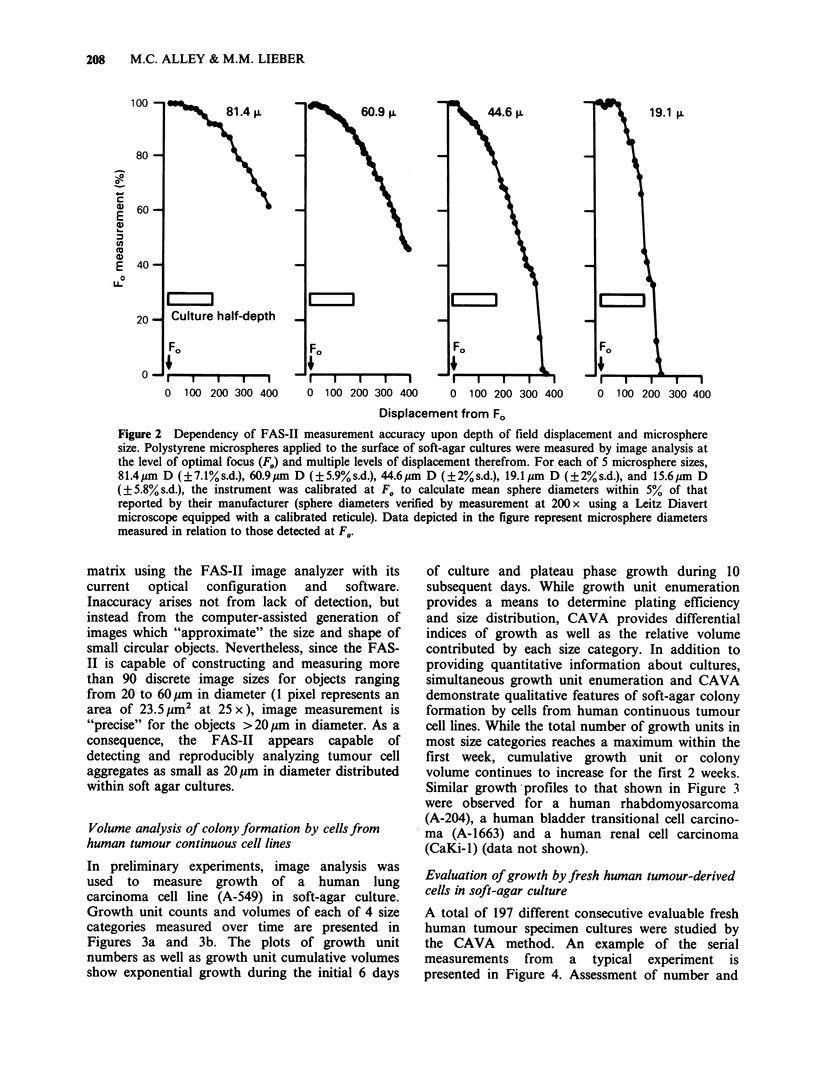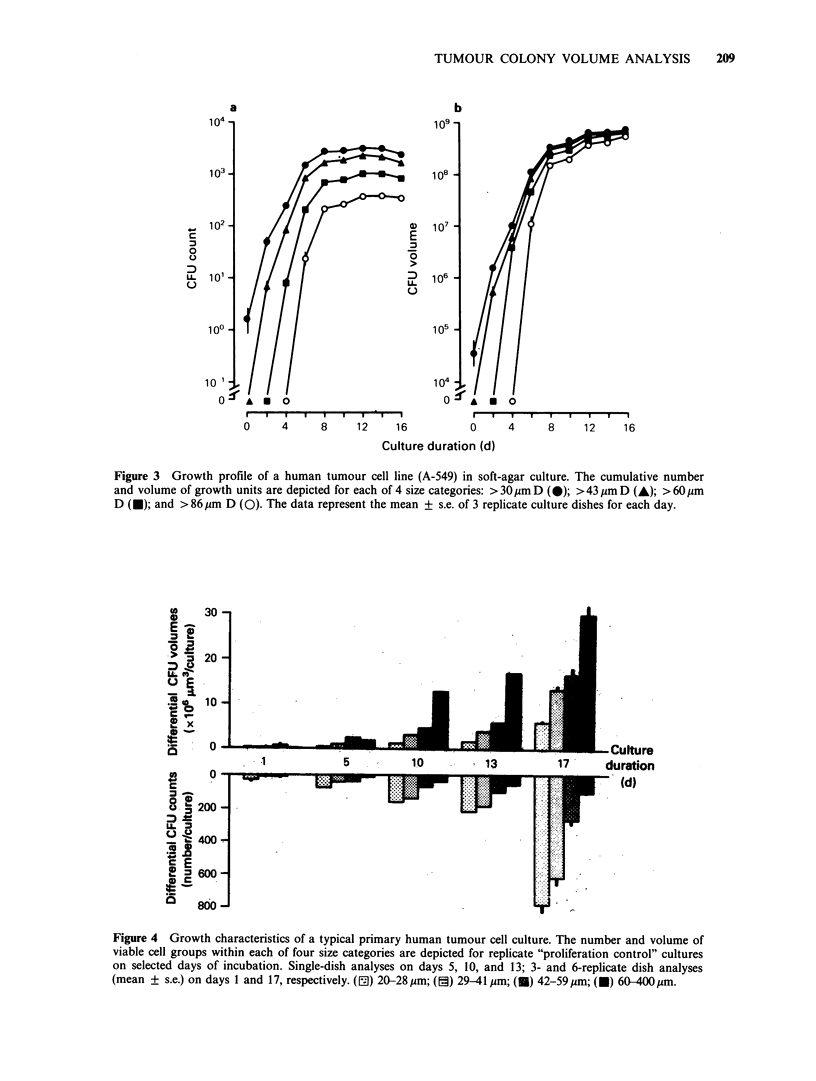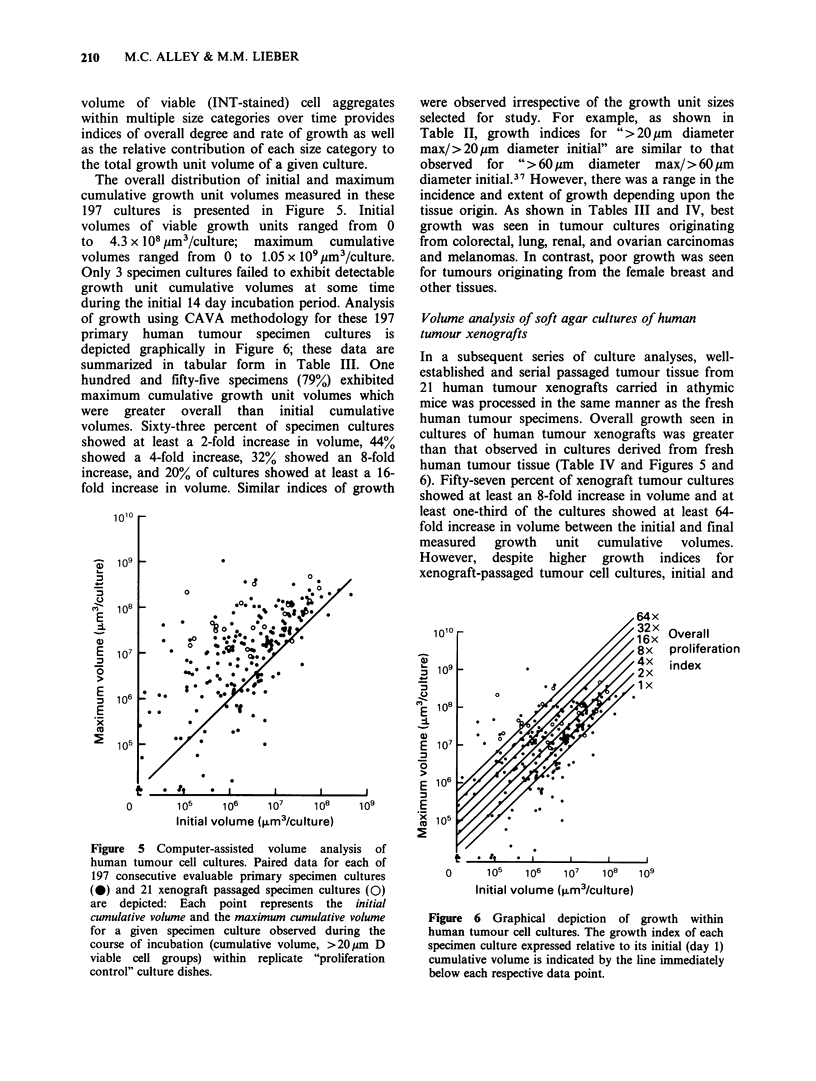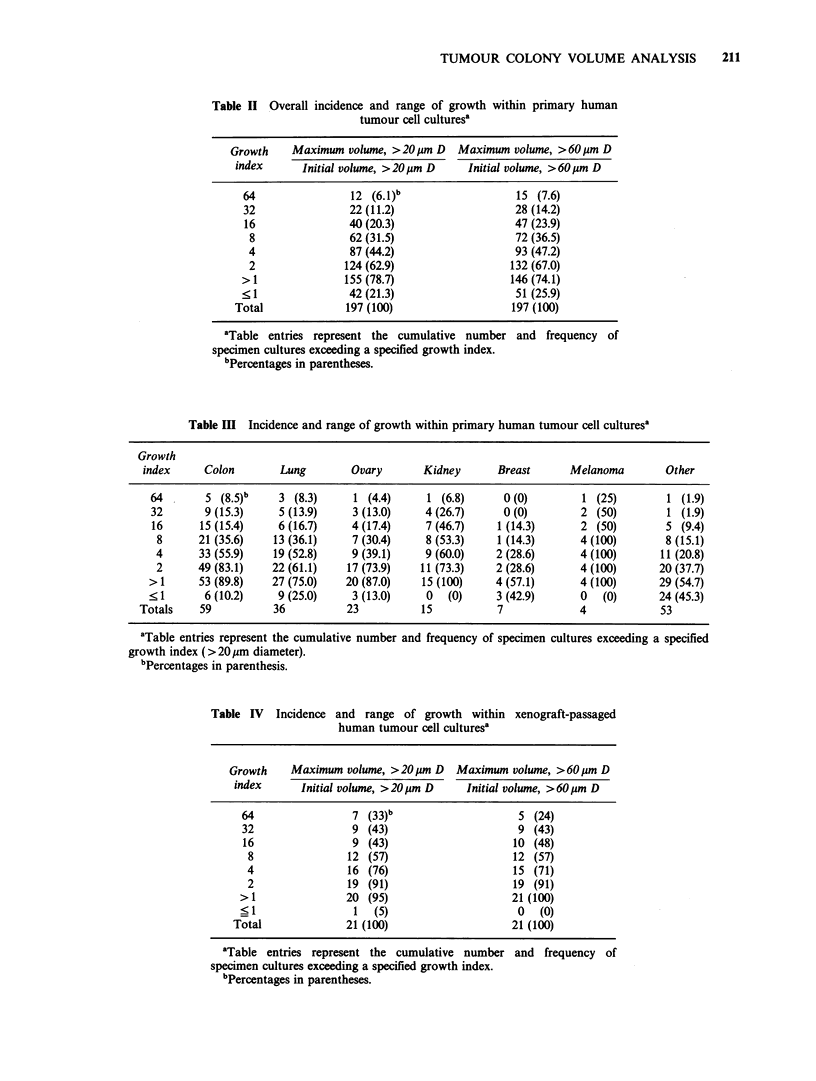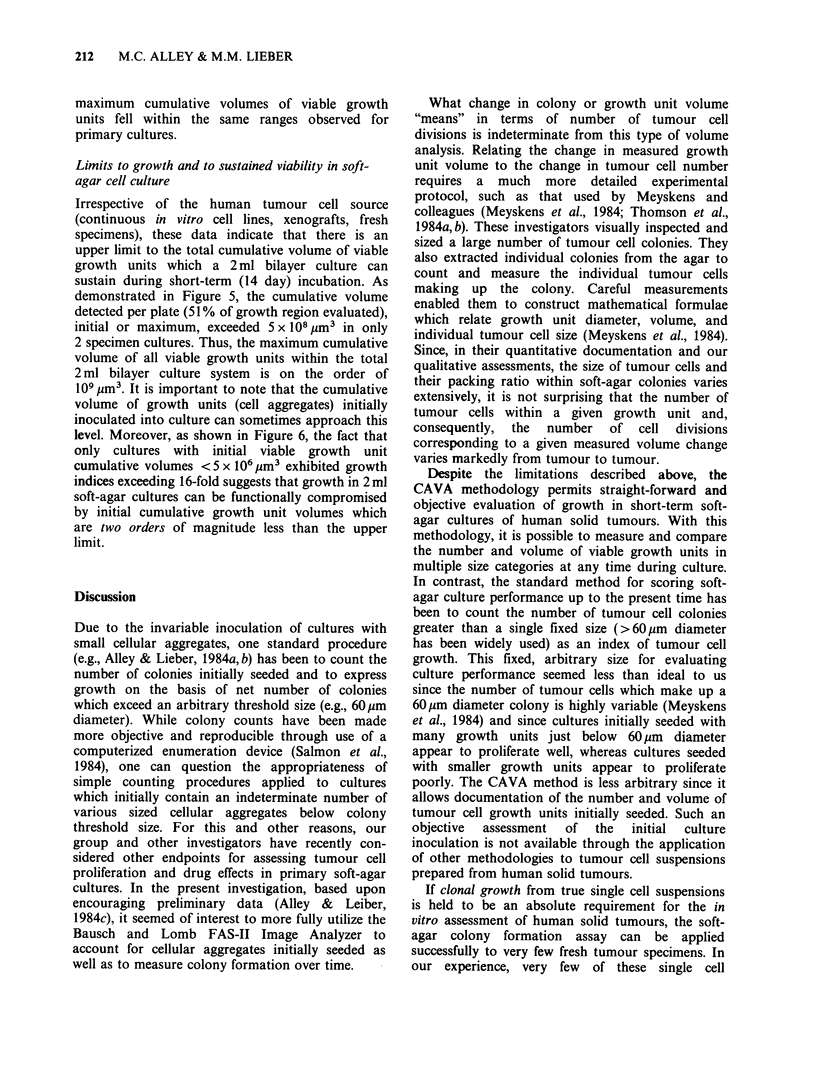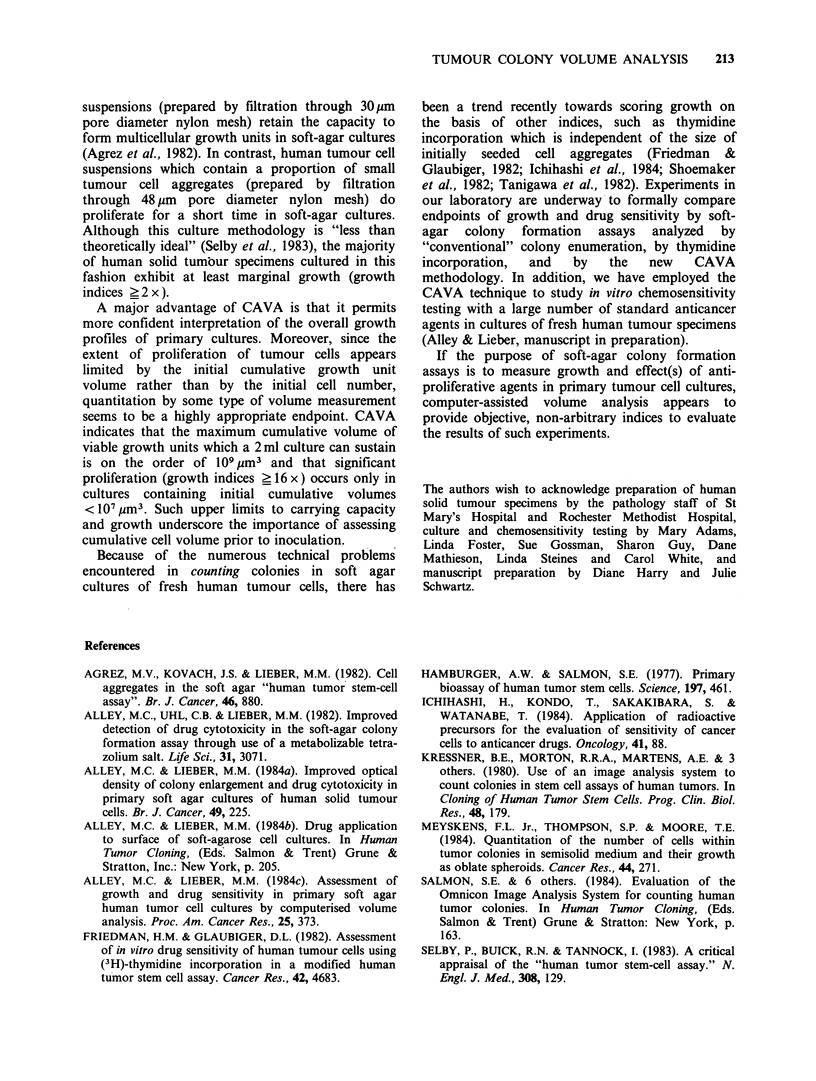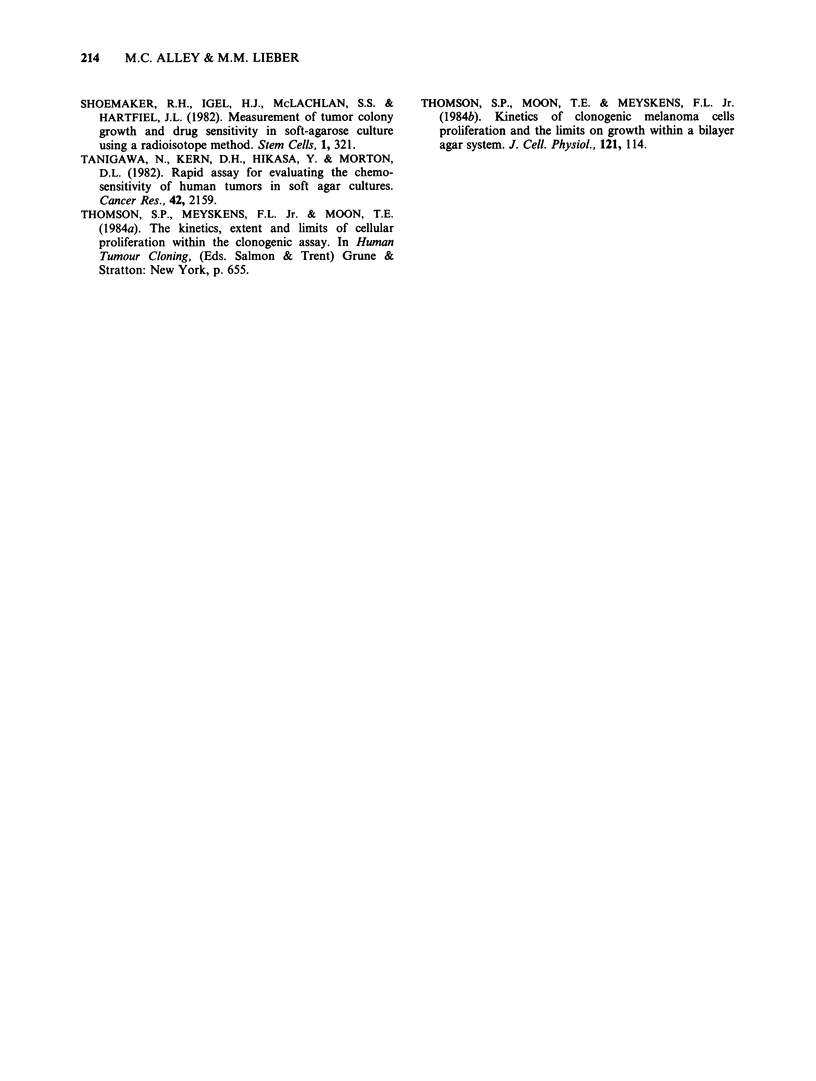# Measurement of human tumour cell growth in soft-agar cultures using computer-assisted volume analysis.

**DOI:** 10.1038/bjc.1985.179

**Published:** 1985-08

**Authors:** M. C. Alley, M. M. Lieber

## Abstract

Growth in soft-agar bilayer cultures of human tumour cells derived from 4 in vitro continuous cell lines, from 21 xenografts carried in athymic mice, and from 197 samples of fresh human solid tumours of various histologic types was analyzed by computer-assisted image analysis. Replicate cultures for each specimen were assessed on successive days of incubation for the number and volume of growth units within multiple size categories. Our results confirm the recent finding of others that there is an upper limit of approximately 10(9) microns 3 to the cumulative growth unit volume obtainable in a 2 ml bilayer soft agar culture system. Since this upper limit to the carrying capacity of the closed culture system exists, the extent of growth within the cultures is determined in a fundamental way by the cumulative volume of growth units initially inoculated into cultures. A growth index of greater than or equal to 16-fold was only seen when initial cumulative growth unit volume was less than 10(7) microns 3 per culture dish. Computer-assisted volume analysis (CAVA) appears to be a useful quantitative method to study the growth of human tumour cells in soft agar cultures.


Br. J. Cancer (1985), 52, 205–214

### Measurement of human tumour cell growth in soft-agar cultures using computer-assisted volume analysis

M.C. Alley* & M.M. Lieber

*Department of Urology, Mayo Clinic, Rochester, MN 55905, USA.*

**Summary** Growth in soft-agar bilayer cultures of human tumour cells derived from 4 *in vitro* continuous cell lines, from 21 xenografts carried in athymic mice, and from 197 samples of fresh human solid tumours of various histologic types was analyzed by computer-assisted image analysis. Replicate cultures for each specimen were assessed on successive days of incubation for the number and volume of growth units within multiple size categories. Our results confirm the recent finding of others that there is an upper limit of $\sim 10^9\,\mu m^3$ to the cumulative growth unit volume obtainable in a 2 ml bilayer soft agar culture system. Since this upper limit to the carrying capacity of the closed culture system exists, the extent of growth within the cultures is determined in a fundamental way by the cumulative volume of growth units initially inoculated into cultures. A growth index of $\geqq 16$-fold was only seen when initial cumulative growth unit volume was $< 10^7\,\mu m^3$ per culture dish. Computer-assisted volume analysis (CAVA) appears to be a useful quantitative method to study the growth of human tumour cells in soft agar cultures.

Single cell suspensions from well-established *in vitro* propagated human tumour cell lines can be readily prepared and generally proliferate well *in vitro*. Clonal growth of such cells in semi-solid culture media (such as 0.3% agar) has been used to study various aspects of tumour cell proliferation and sensitivity to anticancer agents. In contrast, preparation of pure single cell suspensions from fresh human solid malignant neoplasms is difficult and such cell suspensions generally proliferate poorly in soft agar or agarose cultures. Moreover, almost all of the observed growth appears to result from the enlargement of seeded small cell aggregates rather than from the clonal expansion of single cells (Agrez *et al.*, 1982).

Our laboratory at the Mayo Clinic has extensively employed short-term soft agar cultures to assess growth and chemosensitivity of human solid tumour cells. During the past 5 years, we have studied samples from over 6000 different human solid tumours of various histologic types using this assay methodology. We have developed a number of methodologic modifications to eliminate or control for some of the technical problems associated with primary soft agar cultures prepared using the basic Hamburger–Salmon technique (Hamburger & Salmon, 1977; Alley *et al.*, 1982; Alley & Lieber, 1984*a b*). The invariable presence of seeded small tumour cell aggregates when such soft agar cultures are made up using tumour cell suspensions prepared from fresh human solid tumours for us has been the single technical variable which has most seriously complicated assessment of assays of this type.

Counting and sizing the colony forming units or growth units in the bilayer soft agar cultures has been made more objective and reproducible through the use of a dedicated computerized image analysis enumeration system (Salmon *et al.*, 1984). It seemed of interest to more fully utilize the Bausch & Lomb FAS-II Image Analyzer to account more definitively for the number and sizes of cellular aggregates initially seeded as well as the change in growth unit sizes over time. The standard battery of software furnished with the FAS-II instrument contains a program which automatically calculates the diameter, cross-sectional area, and extrapolated volume of individual cell groups (growth units) present within the bilayer soft agar cultures (Kressner *et al.*, 1980). In the studies reported herein, we utilized computer-assisted volume analysis (CAVA) to study the growth of human tumour cell growth units seeded into soft agar culture.

### Materials and methods

### *Tumour acquisition, digestion and filtration*

Methods of tumour acquisition, digestion, and filtration have been described previously (Alley & Lieber, 1984*a*). In brief, minced tumour tissue was incubated in RPMI 1640 medium containing 10% foetal calf serum, 0.6% collagenase II, and 0.002% DNase I for 2 h at 37°C. Disaggregated cells were filtered by gravity or minimal pressure through one or more 100 $\mu m$ pore size filtration units. Cells were

Correspondence: M.M. Lieber
Received 31 January 1985; and in revised form 29 March 1985.
*Current address: NCI Frederick Cancer Research Facility, Frederick, MD 21701, USA.

© The Macmillan Press Ltd., 1985

washed once and resuspended in a small volume of fully supplemented CMRL 1066 medium. Cell suspensions were filtered through a 70 μm pore size filtration unit just prior to microscopic examination. Suspensions exhibiting cellular aggregates >60 μm in diameter were filtered one additional time through a 48 μm pore size filtration unit prior to cell count. Final suspensions for cell culture were prepared only by dilution of previously filtered suspensions. The following primary human tumour types were studied: colectoral carcinoma, 59 specimens; lung carcinoma, 36; ovarian carcinoma, 23; renal cell carcinoma, 15; breast carcinoma, 7; melanoma, 4; miscellaneous tumour types, 53. Human carcinomas were serially transplanted using BALB/c nude athymic mice (Sprague Dawley). The tumours were transplanted s.c. in the flanks. At ~2 cm in diameter tumour size, the nude mouse was sacrificed by cervical dislocation and the tumour was removed aseptically. Subsequent xenograft tumour cell preparation was carried out exactly as described above for primary human tumour material.

*Soft agarose cell culture and culture surface drug application*

While the basal layer of cultures was prepared with 0.5% agar, the cellular (upper) layer was formulated with 0.3% Seaplaque agarose. A single, "bulk" cell suspension was prepared for each specimen culture; inoculation of 1 ml aliquots (500,000 nucleated cells) in replicate dishes was performed with a constant-volume step-syringe. Following agarose gel formation, an aliquot (100 μl) of drug solution or drug vehicle was applied to the surface of each culture dish (Alley & Lieber, 1984*b*).

*Analysis of INT stained cultures*

Cultures were examined periodically with the aid of an inverted stage, phase-light microscope (Leitz Diavert equipped with reticule, Ernst Leitz Co., Rockleigh, NJ) and scored by a computerized image analyzer, the Omnicon Feature Analysis System, Model II (FAS-II, Bausch and Lomb, Inc., Rochester, NY). The "flat" evaluable region of each culture dish (35 contiguous fields equivalent to 51% of the culture volume, away from the meniscus at the edge) was monitored through use of the standard Omnicon colony analysis program. Scoring of cell groups was based upon measurement of cross-sectional area (contiguous array of picture points). Computations of the diameter and volume of individual cell groups assume spherical geometry. Cell groups possessing maximum to minimum center-of-gravity chord ratios exceeding 2.5 were excluded from analysis (Kressner *et al.*, 1980). Cumulative counts and volumes were determined in each of four size categories (>20.5, >29.3, >41.9, >59.9 μm diameter). Instrument calibration utilizing the Omnicon Test Plate 3 was confirmed through use of uniform polystyrene microspheres (nominal sizes: 15, 20, 40, 60, 80 μm diameter; Duke Scientific Corporation, Palo Alto, CA).

Three proliferation control cultures were analyzed on Day 1 of incubation and single proliferation control cultures were analyzed on days 5, 10 and 13. The main body of dishes for a given specimen culture was analyzed as soon as significant growth was observed to occur in the proliferation control cultures (usually 5–17 days). Culture dishes were stained with a metabolizable, vital dye, INT [2-(p-iodophenyl)-3-(p-nitrophenyl)-5-phenyl tetrazolium chloride], for 24 h just prior to analysis (Alley *et al.*, 1982). Selective scoring of viable cell groups by the image analyzer was achieved by adjustment of the instrument detection threshold (minimum optical density) to exclude images of non-stained cell groups and debris.

### Results

*Accuracy and precision of image measurements*

In order to assess the capability of the FAS-II image analyzer to measure the small roughly-spherical objects known to be present in primary soft agar cultures, a number of preliminary experiments were performed. Computerized image analysis measurements of the areas of small circles are presented in Table I. In general, the instrument accurately measured the area of circles 50 μm in diameter or larger at all levels of magnification. Accuracy in measuring image areas of objects <50 μm in diameter appeared limited at lower levels of magnification (25× and 40× objectives) by the ability of the instrument to construct "computer images" whose shape approximated to that of circular objects using contiguous points (pixels) of rectangular shape. Since the relative spatial configuration of pixels is fixed and since the area represented by individual pixels varies inversely with the degree of magnification, it was not surprising that a disparity was found between the actual and measured dimensions when the area of an individual pixel represented a significant proportion of the total image area. Thus, the accuracy of computer-assisted image analysis with this instrument is limited by the process constructing and then measuring the areas of small images at low power magnification.

Measurement of colony (or growth unit) size in soft-agar cultures also requires assessment of limitations imposed by culture thickness. A 1 ml layer of cell suspension in a 35 ml culture dish

**Table I** Accuracy of FAS-II measurements in a single plane

| *Actual circle dimensions*[a] | | *Area measured by image analysis*[b] | | |
|---|---|---|---|---|
| *Diameter* (*μm*) | *Area* (*μm*$^2$) | *25×* | *40×* | *100×* |
| 10.0 | 78.54 | — | 46.9±20 (59.7) | 78.1±4.3 (99.4) |
| 25.0 | 490.9 | 341±25 (69.5) | 495±9 (101) | 492±8 (100) |
| 50.0 | 1,964 | 1,904±26 (97.0) | 1,988±33 (101) | 2,011±6 (102) |
| 100 | 7,854 | 7,857±59 (100) | 7,866±40 (100) | 7,853±20 (100) |
| 250 | 49,090 | 53,290±136 (109) | 50,460±67 (103) | 49,107±29 (100) |
| 500 | 196,400 | 216,445±161 (110) | 201,950±79 (103) | 188,368±32 (95.9) |
| 1000 | 785,400 | 869,800±113 (111) | 807,849±96 (103) | — |

[a]Electroplated circles of multiple sizes on the surface of a glass calibration slide, Omnicon Test Plate 3 (Bausch and Lomb, Rochester, NY).
[b]Table entries are the mean ± s.d. of 4 determinations and (percent of actual circle dimension) for each of 3 levels of magnification (25×, 40×, 100×). Instrument calibration was performed using the 100 μm diameter circle as the primary standard for all levels of magnification.

produces a circular disc ~1 mm thick (1,040 μm by calculation). To quantitate the effect of culture thickness on the accuracy of colony size estimates, polystyrene microspheres of known size applied to the surface of soft-agar cultures were measured over a range of displacements from the optimal focal plane ($F_o$) of each of three microscope objective lenses. As shown in Figure 1, the accuracy of measuring 60 μm diameter spheres was not significantly affected by $F_o$ displacements encountered in soft-agar culture when the low power objective (25× magnification) was employed; measurements were within 90% of true diameter at a displacement of 520 μm (the 1 ml volume half-depth). Substantially greater measurement errors were associated with use of the higher power (40× and 100×) objectives which had much narrower depths of field. In a subsequent experiment, measurement accuracy was determined for a range of microsphere sizes at the low power (25× objective) magnification (routinely used for serial measurements of growth in soft-agar cultures). As shown in Figure 2, a range of detection efficiencies was observed. While 15.5 μm diameter spheres were inefficiently detected at $F_o$ displacements >200 μm (data not shown), spheres 19.1 μm in diameter and greater were efficiently detected at $F_o$ displacements up to 520 μm.

The data in Table I, Figure 1, and Figure 2 demonstrate the limitations of measurement accuracy for 3-dimensional objects in soft-agar

**Figure 1** Dependency of FAS-II measurement accuracy upon degree of magnification and object depth-of-field displacement. Polystyrene microspheres, 60.9 μm D (±5.9% s.d.), applied to the surface of soft-agar culture were measured by image analysis at the plane of optimal focus ($F_o$) as well as multiple levels of displacement from $F_o$. For each degree of magnification the instrument was calibrated at $F_o$ to calculate mean diameters within 2% of that reported by the manufacturer. (□) 100×; (▲) 40×; (●) 25×.

**Figure 2** Dependency of FAS-II measurement accuracy upon depth of field displacement and microsphere size. Polystyrene microspheres applied to the surface of soft-agar cultures were measured by image analysis at the level of optimal focus ($F_o$) and multiple levels of displacement therefrom. For each of 5 microsphere sizes, 81.4 μm D (±7.1% s.d.), 60.9 μm D (±5.9% s.d.), 44.6 μm D (±2% s.d.), 19.1 μm D (±2% s.d.), and 15.6 μm D (±5.8% s.d.), the instrument was calibrated at $F_o$ to calculate mean sphere diameters within 5% of that reported by their manufacturer (sphere diameters verified by measurement at 200× using a Leitz Diavert microscope equipped with a calibrated reticule). Data depicted in the figure represent microsphere diameters measured in relation to those detected at $F_o$.

matrix using the FAS-II image analyzer with its current optical configuration and software. Inaccuracy arises not from lack of detection, but instead from the computer-assisted generation of images which "approximate" the size and shape of small circular objects. Nevertheless, since the FAS-II is capable of constructing and measuring more than 90 discrete image sizes for objects ranging from 20 to 60 μm in diameter (1 pixel represents an area of 23.5 μm$^2$ at 25×), image measurement is "precise" for the objects >20 μm in diameter. As a consequence, the FAS-II appears capable of detecting and reproducibly analyzing tumour cell aggregates as small as 20 μm in diameter distributed within soft agar cultures.

### *Volume analysis of colony formation by cells from human tumour continuous cell lines*

In preliminary experiments, image analysis was used to measure growth of a human lung carcinoma cell line (A-549) in soft-agar culture. Growth unit counts and volumes of each of 4 size categories measured over time are presented in Figures 3a and 3b. The plots of growth unit numbers as well as growth unit cumulative volumes show exponential growth during the initial 6 days of culture and plateau phase growth during 10 subsequent days. While growth unit enumeration provides a means to determine plating efficiency and size distribution, CAVA provides differential indices of growth as well as the relative volume contributed by each size category. In addition to providing quantitative information about cultures, simultaneous growth unit enumeration and CAVA demonstrate qualitative features of soft-agar colony formation by cells from human continuous tumour cell lines. While the total number of growth units in most size categories reaches a maximum within the first week, cumulative growth unit or colony volume continues to increase for the first 2 weeks. Similar growth profiles to that shown in Figure 3 were observed for a human rhabdomyosarcoma (A-204), a human bladder transitional cell carcinoma (A-1663) and a human renal cell carcinoma (CaKi-1) (data not shown).

### *Evaluation of growth by fresh human tumour-derived cells in soft-agar culture*

A total of 197 different consecutive evaluable fresh human tumour specimen cultures were studied by the CAVA method. An example of the serial measurements from a typical experiment is presented in Figure 4. Assessment of number and

**Figure 3** Growth profile of a human tumour cell line (A-549) in soft-agar culture. The cumulative number and volume of growth units are depicted for each of 4 size categories: >30 μm D (●); >43 μm D (▲); >60 μm D (■); and >86 μm D (○). The data represent the mean ± s.e. of 3 replicate culture dishes for each day.

**Figure 4** Growth characteristics of a typical primary human tumour cell culture. The number and volume of viable cell groups within each of four size categories are depicted for replicate "proliferation control" cultures on selected days of incubation. Single-dish analyses on days 5, 10, and 13; 3- and 6-replicate dish analyses (mean ± s.e.) on days 1 and 17, respectively. (▫) 20–28 μm; (▤) 29–41 μm; (▩) 42–59 μm; (■) 60–400 μm.

volume of viable (INT-stained) cell aggregates within multiple size categories over time provides indices of overall degree and rate of growth as well as the relative contribution of each size category to the total growth unit volume of a given culture.

The overall distribution of initial and maximum cumulative growth unit volumes measured in these 197 cultures is presented in Figure 5. Initial volumes of viable growth units ranged from 0 to $4.3 \times 10^8\,\mu m^3$/culture; maximum cumulative volumes ranged from 0 to $1.05 \times 10^9\,\mu m^3$/culture. Only 3 specimen cultures failed to exhibit detectable growth unit cumulative volumes at some time during the initial 14 day incubation period. Analysis of growth using CAVA methodology for these 197 primary human tumour specimen cultures is depicted graphically in Figure 6; these data are summarized in tabular form in Table III. One hundred and fifty-five specimens (79%) exhibited maximum cumulative growth unit volumes which were greater overall than initial cumulative volumes. Sixty-three percent of specimen cultures showed at least a 2-fold increase in volume, 44% showed a 4-fold increase, 32% showed an 8-fold increase, and 20% of cultures showed at least a 16-fold increase in volume. Similar indices of growth were observed irrespective of the growth unit sizes selected for study. For example, as shown in Table II, growth indices for "$>20\,\mu m$ diameter max/$>20\,\mu m$ diameter initial" are similar to that observed for "$>60\,\mu m$ diameter max/$>60\,\mu m$ diameter initial.[37] However, there was a range in the incidence and extent of growth depending upon the tissue origin. As shown in Tables III and IV, best growth was seen in tumour cultures originating from colorectal, lung, renal, and ovarian carcinomas and melanomas. In contrast, poor growth was seen for tumours originating from the female breast and other tissues.

**Figure 5** Computer-assisted volume analysis of human tumour cell cultures. Paired data for each of 197 consecutive evaluable primary specimen cultures (●) and 21 xenograft passaged specimen cultures (○) are depicted: Each point represents the *initial cumulative volume* and the *maximum cumulative volume* for a given specimen culture observed during the course of incubation (cumulative volume, $>20\,\mu m$ D viable cell groups) within replicate "proliferation control" culture dishes.

*Volume analysis of soft agar cultures of human tumour xenografts*

In a subsequent series of culture analyses, well-established and serial passaged tumour tissue from 21 human tumour xenografts carried in athymic mice was processed in the same manner as the fresh human tumour specimens. Overall growth seen in cultures of human tumour xenografts was greater than that observed in cultures derived from fresh human tumour tissue (Table IV and Figures 5 and 6). Fifty-seven percent of xenograft tumour cultures showed at least an 8-fold increase in volume and at least one-third of the cultures showed at least 64-fold increase in volume between the initial and final measured growth unit cumulative volumes. However, despite higher growth indices for xenograft-passaged tumour cell cultures, initial and

**Figure 6** Graphical depiction of growth within human tumour cell cultures. The growth index of each specimen culture expressed relative to its initial (day 1) cumulative volume is indicated by the line immediately below each respective data point.

**Table II** Overall incidence and range of growth within primary human tumour cell cultures[a]

| Growth index | Maximum volume, >20 μm D / Initial volume, >20 μm D | Maximum volume, >60 μm D / Initial volume, >60 μm D |
|---|---|---|
| 64 | 12 (6.1)[b] | 15 (7.6) |
| 32 | 22 (11.2) | 28 (14.2) |
| 16 | 40 (20.3) | 47 (23.9) |
| 8 | 62 (31.5) | 72 (36.5) |
| 4 | 87 (44.2) | 93 (47.2) |
| 2 | 124 (62.9) | 132 (67.0) |
| >1 | 155 (78.7) | 146 (74.1) |
| ≤1 | 42 (21.3) | 51 (25.9) |
| Total | 197 (100) | 197 (100) |

[a]Table entries represent the cumulative number and frequency of specimen cultures exceeding a specified growth index.
[b]Percentages in parentheses.

**Table III** Incidence and range of growth within primary human tumour cell cultures[a]

| Growth index | Colon | Lung | Ovary | Kidney | Breast | Melanoma | Other |
|---|---|---|---|---|---|---|---|
| 64 | 5 (8.5)[b] | 3 (8.3) | 1 (4.4) | 1 (6.8) | 0 (0) | 1 (25) | 1 (1.9) |
| 32 | 9 (15.3) | 5 (13.9) | 3 (13.0) | 4 (26.7) | 0 (0) | 2 (50) | 1 (1.9) |
| 16 | 15 (15.4) | 6 (16.7) | 4 (17.4) | 7 (46.7) | 1 (14.3) | 2 (50) | 5 (9.4) |
| 8 | 21 (35.6) | 13 (36.1) | 7 (30.4) | 8 (53.3) | 1 (14.3) | 4 (100) | 8 (15.1) |
| 4 | 33 (55.9) | 19 (52.8) | 9 (39.1) | 9 (60.0) | 2 (28.6) | 4 (100) | 11 (20.8) |
| 2 | 49 (83.1) | 22 (61.1) | 17 (73.9) | 11 (73.3) | 2 (28.6) | 4 (100) | 20 (37.7) |
| >1 | 53 (89.8) | 27 (75.0) | 20 (87.0) | 15 (100) | 4 (57.1) | 4 (100) | 29 (54.7) |
| ≤1 | 6 (10.2) | 9 (25.0) | 3 (13.0) | 0 (0) | 3 (42.9) | 0 (0) | 24 (45.3) |
| Totals | 59 | 36 | 23 | 15 | 7 | 4 | 53 |

[a]Table entries represent the cumulative number and frequency of specimen cultures exceeding a specified growth index (>20 μm diameter).
[b]Percentages in parenthesis.

**Table IV** Incidence and range of growth within xenograft-passaged human tumour cell cultures[a]

| Growth index | Maximum volume, >20 μm D / Initial volume, >20 μm D | Maximum volume, >60 μm D / Initial volume, >60 μm D |
|---|---|---|
| 64 | 7 (33)[b] | 5 (24) |
| 32 | 9 (43) | 9 (43) |
| 16 | 9 (43) | 10 (48) |
| 8 | 12 (57) | 12 (57) |
| 4 | 16 (76) | 15 (71) |
| 2 | 19 (91) | 19 (91) |
| >1 | 20 (95) | 21 (100) |
| ≤1 | 1 (5) | 0 (0) |
| Total | 21 (100) | 21 (100) |

[a]Table entries represent the cumulative number and frequency of specimen cultures exceeding a specified growth index.
[b]Percentages in parentheses.

maximum cumulative volumes of viable growth units fell within the same ranges observed for primary cultures.

*Limits to growth and to sustained viability in soft-agar cell culture*

Irrespective of the human tumour cell source (continuous *in vitro* cell lines, xenografts, fresh specimens), these data indicate that there is an upper limit to the total cumulative volume of viable growth units which a 2 ml bilayer culture can sustain during short-term (14 day) incubation. As demonstrated in Figure 5, the cumulative volume detected per plate (51% of growth region evaluated), initial or maximum, exceeded $5 \times 10^8 \mu m^3$ in only 2 specimen cultures. Thus, the maximum cumulative volume of all viable growth units within the total 2 ml bilayer culture system is on the order of $10^9 \mu m^3$. It is important to note that the cumulative volume of growth units (cell aggregates) initially inoculated into culture can sometimes approach this level. Moreover, as shown in Figure 6, the fact that only cultures with initial viable growth unit cumulative volumes $< 5 \times 10^6 \mu m^3$ exhibited growth indices exceeding 16-fold suggests that growth in 2 ml soft-agar cultures can be functionally compromised by initial cumulative growth unit volumes which are *two orders* of magnitude less than the upper limit.

### Discussion

Due to the invariable inoculation of cultures with small cellular aggregates, one standard procedure (e.g., Alley & Lieber, 1984*a, b*) has been to count the number of colonies initially seeded and to express growth on the basis of net number of colonies which exceed an arbitrary threshold size (e.g., 60 $\mu$m diameter). While colony counts have been made more objective and reproducible through use of a computerized enumeration device (Salmon *et al.*, 1984), one can question the appropriateness of simple counting procedures applied to cultures which initially contain an indeterminate number of various sized cellular aggregates below colony threshold size. For this and other reasons, our group and other investigators have recently considered other endpoints for assessing tumour cell proliferation and drug effects in primary soft-agar cultures. In the present investigation, based upon encouraging preliminary data (Alley & Leiber, 1984*c*), it seemed of interest to more fully utilize the Bausch and Lomb FAS-II Image Analyzer to account for cellular aggregates initially seeded as well as to measure colony formation over time.

What change in colony or growth unit volume "means" in terms of number of tumour cell divisions is indeterminate from this type of volume analysis. Relating the change in measured growth unit volume to the change in tumour cell number requires a much more detailed experimental protocol, such as that used by Meyskens and colleagues (Meyskens *et al.*, 1984; Thomson *et al.*, 1984*a, b*). These investigators visually inspected and sized a large number of tumour cell colonies. They also extracted individual colonies from the agar to count and measure the individual tumour cells making up the colony. Careful measurements enabled them to construct mathematical formulae which relate growth unit diameter, volume, and individual tumour cell size (Meyskens *et al.*, 1984). Since, in their quantitative documentation and our qualitative assessments, the size of tumour cells and their packing ratio within soft-agar colonies varies extensively, it is not surprising that the number of tumour cells within a given growth unit and, consequently, the number of cell divisions corresponding to a given measured volume change varies markedly from tumour to tumour.

Despite the limitations described above, the CAVA methodology permits straight-forward and objective evaluation of growth in short-term soft-agar cultures of human solid tumours. With this methodology, it is possible to measure and compare the number and volume of viable growth units in multiple size categories at any time during culture. In contrast, the standard method for scoring soft-agar culture performance up to the present time has been to count the number of tumour cell colonies greater than a single fixed size (>60 $\mu$m diameter has been widely used) as an index of tumour cell growth. This fixed, arbitrary size for evaluating culture performance seemed less than ideal to us since the number of tumour cells which make up a 60 $\mu$m diameter colony is highly variable (Meyskens *et al.*, 1984) and since cultures initially seeded with many growth units just below 60 $\mu$m diameter appear to proliferate well, whereas cultures seeded with smaller growth units appear to proliferate poorly. The CAVA method is less arbitrary since it allows documentation of the number and volume of tumour cell growth units initially seeded. Such an objective assessment of the initial culture inoculation is not available through the application of other methodologies to tumour cell suspensions prepared from human solid tumours.

If *clonal growth* from true single cell suspensions is held to be an absolute requirement for the *in vitro* assessment of human solid tumours, the soft-agar colony formation assay can be applied successfully to very few fresh tumour specimens. In our experience, very few of these single cell suspensions (prepared by filtration through 30 μm pore diameter nylon mesh) retain the capacity to form multicellular growth units in soft-agar cultures (Agrez *et al.*, 1982). In contrast, human tumour cell suspensions which contain a proportion of small tumour cell aggregates (prepared by filtration through 48 μm pore diameter nylon mesh) do proliferate for a short time in soft-agar cultures. Although this culture methodology is "less than theoretically ideal" (Selby *et al.*, 1983), the majority of human solid tumour specimens cultured in this fashion exhibit at least marginal growth (growth indices $\geqq 2\times$).

A major advantage of CAVA is that it permits more confident interpretation of the overall growth profiles of primary cultures. Moreover, since the extent of proliferation of tumour cells appears limited by the initial cumulative growth unit volume rather than by the initial cell number, quantitation by some type of volume measurement seems to be a highly appropriate endpoint. CAVA indicates that the maximum cumulative volume of viable growth units which a 2 ml culture can sustain is on the order of $10^9\,\mu m^3$ and that significant proliferation (growth indices $\geqq 16\times$) occurs only in cultures containing initial cumulative volumes $< 10^7\,\mu m^3$. Such upper limits to carrying capacity and growth underscore the importance of assessing cumulative cell volume prior to inoculation.

Because of the numerous technical problems encountered in *counting* colonies in soft agar cultures of fresh human tumour cells, there has been a trend recently towards scoring growth on the basis of other indices, such as thymidine incorporation which is independent of the size of initially seeded cell aggregates (Friedman & Glaubiger, 1982; Ichihashi *et al.*, 1984; Shoemaker *et al.*, 1982; Tanigawa *et al.*, 1982). Experiments in our laboratory are underway to formally compare endpoints of growth and drug sensitivity by soft-agar colony formation assays analyzed by "conventional" colony enumeration, by thymidine incorporation, and by the new CAVA methodology. In addition, we have employed the CAVA technique to study *in vitro* chemosensitivity testing with a large number of standard anticancer agents in cultures of fresh human tumour specimens (Alley & Lieber, manuscript in preparation).

If the purpose of soft-agar colony formation assays is to measure growth and effect(s) of anti-proliferative agents in primary tumour cell cultures, computer-assisted volume analysis appears to provide objective, non-arbitrary indices to evaluate the results of such experiments.

The authors wish to acknowledge preparation of human solid tumour specimens by the pathology staff of St Mary's Hospital and Rochester Methodist Hospital, culture and chemosensitivity testing by Mary Adams, Linda Foster, Sue Gossman, Sharon Guy, Dane Mathieson, Linda Steines and Carol White, and manuscript preparation by Diane Harry and Julie Schwartz.

**References**

AGREZ, M.V., KOVACH, J.S. & LIEBER, M.M. (1982). Cell aggregates in the soft agar "human tumor stem-cell assay". *Br. J. Cancer*, **46**, 880.

ALLEY, M.C., UHL, C.B. & LIEBER, M.M. (1982). Improved detection of drug cytotoxicity in the soft-agar colony formation assay through use of a metabolizable tetrazolium salt. *Life Sci.*, **31**, 3071.

ALLEY, M.C. & LIEBER, M.M. (1984*a*). Improved optical density of colony enlargement and drug cytotoxicity in primary soft agar cultures of human solid tumour cells. *Br. J. Cancer*, **49**, 225.

ALLEY, M.C. & LIEBER, M.M. (1984*b*). Drug application to surface of soft-agarose cell cultures. In *Human Tumor Cloning*, (Eds. Salmon & Trent) Grune & Stratton, Inc.: New York, p. 205.

ALLEY, M.C. & LIEBER, M.M. (1984*c*). Assessment of growth and drug sensitivity in primary soft agar human tumor cell cultures by computerised volume analysis. *Proc. Am. Cancer Res.*, **25**, 373.

FRIEDMAN, H.M. & GLAUBIGER, D.L. (1982). Assessment of *in vitro* drug sensitivity of human tumour cells using ($^3$H)-thymidine incorporation in a modified human tumor stem cell assay. *Cancer Res.*, **42**, 4683.

HAMBURGER, A.W. & SALMON, S.E. (1977). Primary bioassay of human tumor stem cells. *Science*, **197**, 461.

ICHIHASHI, H., KONDO, T., SAKAKIBARA, S. & WATANABE, T. (1984). Application of radioactive precursors for the evaluation of sensitivity of cancer cells to anticancer drugs. *Oncology*, **41**, 88.

KRESSNER, B.E., MORTON, R.R.A., MARTENS, A.E. & 3 others. (1980). Use of an image analysis system to count colonies in stem cell assays of human tumors. In *Cloning of Human Tumor Stem Cells. Prog. Clin. Biol. Res.*, **48**, 179.

MEYSKENS, F.L. Jr., THOMPSON, S.P. & MOORE, T.E. (1984). Quantitation of the number of cells within tumor colonies in semisolid medium and their growth as oblate spheroids. *Cancer Res.*, **44**, 271.

SALMON, S.E. & 6 others. (1984). Evaluation of the Omnicon Image Analysis System for counting human tumor colonies. In *Human Tumor Cloning*, (Eds. Salmon & Trent) Grune & Stratton: New York, p. 163.

SELBY, P., BUICK, R.N. & TANNOCK, I. (1983). A critical appraisal of the "human tumor stem-cell assay." *N. Engl. J. Med.*, **308**, 129.

SHOEMAKER, R.H., IGEL, H.J., McLACHLAN, S.S. & HARTFIEL, J.L. (1982). Measurement of tumor colony growth and drug sensitivity in soft-agarose culture using a radioisotope method. *Stem Cells*, **1**, 321.

TANIGAWA, N., KERN, D.H., HIKASA, Y. & MORTON, D.L. (1982). Rapid assay for evaluating the chemosensitivity of human tumors in soft agar cultures. *Cancer Res.*, **42**, 2159.

THOMSON, S.P., MEYSKENS, F.L. Jr. & MOON, T.E. (1984*a*). The kinetics, extent and limits of cellular proliferation within the clonogenic assay. In *Human Tumour Cloning*, (Eds. Salmon & Trent) Grune & Stratton: New York, p. 655.

THOMSON, S.P., MOON, T.E. & MEYSKENS, F.L. Jr. (1984*b*). Kinetics of clonogenic melanoma cells proliferation and the limits on growth within a bilayer agar system. *J. Cell. Physiol.*, **121**, 114.